# Hybrid Threshold Denoising Framework Using Singular Value Decomposition for Side-Channel Analysis Preprocessing

**DOI:** 10.3390/e25081133

**Published:** 2023-07-28

**Authors:** Yuanzhen Wang, Hongxin Zhang, Xing Fang, Xiaotong Cui, Wenxu Ning, Danzhi Wang, Fan Fan, Lei Shu

**Affiliations:** 1 School of Cyberspace Security, Beijing University of Posts and Telecommunications, Beijing 100876, China; wangyz7@bupt.edu.cn (Y.W.); ningwenxu@bupt.edu.cn (W.N.); 2 School of Electronic Engineering, Beijing University of Posts and Telecommunications, Beijing 100876, China; fancy_t@bupt.edu.cn (X.F.); cuixiaotong@bupt.edu.cn (X.C.); danzhiwang@bupt.edu.cn (D.W.); 3 Beijing Key Laboratory of Work Safety Intelligent Monitoring, Beijing University of Posts and Telecommunications, Beijing 100876, China; 4 Beijing Microelectronics Technology Institute, Beijing 100076, China; 18911650922@163.com (F.F.); 18001350960@163.com (L.S.)

**Keywords:** side-channel analysis, signal preprocessing, Schatten norm, low-rank matrix approximation, singular value decomposition, noise filtering

## Abstract

The traces used in side-channel analysis are essential to breaking the key of encryption and the signal quality greatly affects the correct rate of key guessing. Therefore, the preprocessing of side-channel traces plays an important role in side-channel analysis. The process of side-channel leakage signal acquisition is usually affected by internal circuit noise, external environmental noise, and other factors, so the collected signal is often mixed with strong noise. In order to extract the feature information of side-channel signals from very low signal-to-noise ratio traces, a hybrid threshold denoising framework using singular value decomposition is proposed for side-channel analysis preprocessing. This framework is based on singular value decomposition and introduces low-rank matrix approximation theory to improve the rank selection methods of singular value decomposition. This paper combines the hard threshold method of truncated singular value decomposition with the soft threshold method of singular value shrinkage damping and proposes a hybrid threshold denoising framework using singular value decomposition for the data preprocessing step of side-channel analysis as a general preprocessing method for non-profiled side-channel analysis. The data used in the experimental evaluation are from the raw traces of the public database of DPA contest V2 and AES_HD. The success rate curve of non-profiled side-channel analysis further confirms the effectiveness of the proposed framework. Moreover, the signal-to-noise ratio of traces is significantly improved after preprocessing, and the correlation with the correct key is also significantly enhanced. Experimental results on DPA v2 and AES_HD show that the proposed noise reduction framework can be effectively applied to the side-channel analysis preprocessing step, and can successfully improve the signal-to-noise ratio of the traces and the attack efficiency.

## 1. Introduction

Since the conception of side-channel analysis (SCA), researchers have proposed several side-channel attack methods for different cryptographic devices and encryption algorithms [1]. However, the collected side-channel information includes not only the physical leakage caused by encryption and decryption operations, but also a large number of other unrelated operations, in addition to the interference caused by complex environments. These disturbances are usually treated as noise. Therefore, the process of side-channel signal acquisition is usually strongly interfered with by noise, and the traces preprocessing is very essential [2]. Side-channel preprocessing can remove noise interference and enhance signal characteristics, thereby reducing the computational complexity of side-channel distinguishers and improving the attack efficiency of side-channel analysis [3]. How to effectively extract the feature information from the side-channel signal is the key to improving the signal-to-noise ratio and the success rate of attacks or an attack.

To tackle this issue, a traditional preprocessing method for side-channel analysis can simply average the traces for noise reduction, but this often requires a massive number of traces. Le et al. [4] proposed a preprocessing method for side-channel analysis based on fourth-order cumulants, which used the Gaussian characteristics of noise and the non-Gaussian characteristics of signal to reduce the impact of noise coupled to the traces of the side-channel. Compared with the average noise reduction method, the above method significantly reduces the number of traces required to recover the key, but the calculation is still large. Souissi et al. [5] proposed a new technique based on the Kalman theory, which improves the attack efficiency of power analysis by reducing the traces required to break the key, and proved that Kalman filtering (KF) is more powerful than higher-order statistical (HOS) techniques. Liu et al. [6] proposed a new method to reduce the effect of noise using wavelet analysis to improve the performance of Correlation Power Analysis (CPA), and proved that the noise reduction effect of wavelet transform (WT) is better than that of higher-order cumulants. Feng et al. [7] proposed a side-channel analysis noise reduction pre-processing method based on empirical mode decomposition (EMD), and studied the effectiveness of this method in filtering out high-frequency noise in the traces. At the CHES2015 conference, Santos et al. [8] proposed a pretreatment method of blind source separation based on singular spectrum analysis (SSA). Although the method can extract different characteristic components of the traces through singular value decomposition (SVD), only one component is selected as the signal component, and the signal components in other components are discarded. Sun et al. [9] proposed an improved singular value decomposition method combining Z-score and SVD to extract the raw trace features, so as to improve the attack efficiency of CPA. Ai et al. [10] proposed an improved wavelet transform (WT) method using SSA and detrended fluctuation analysis (DFA), which effectively solved some problems existing in WT noise reduction, but detrended fluctuation would lose some signal features. Gan et al. [11] proposed an improved empirical mode decomposition method, which did not recombine the intrinsic mode functions (IMFs) of the signal after decomposition, but extracted certain IMFs as the new feature signal of CPA. Cheng et al. [12] proposed a hybrid noise reduction method, which firstly used the Butterworth low-pass filter to preprocess the traces, then used EMD to decompose it, and finally used the WT denoising method to remove the noise in the high-frequency part, but the processing process was relatively complex. The related works are summarized in Table 1.

**Figure 1:**
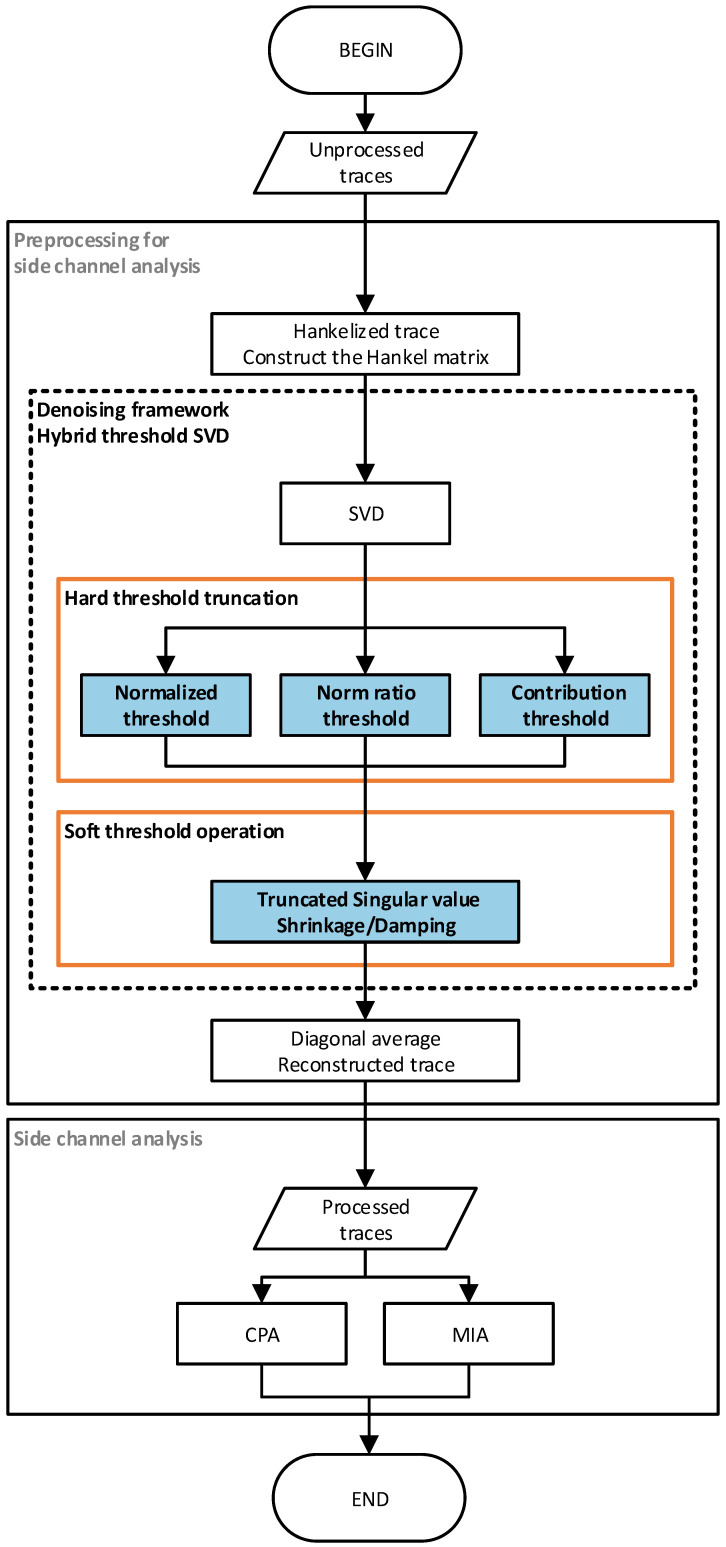
Side-channel attack flow diagram based on singular value decomposition mixed threshold denoising preprocessing method.

In order to solve the problem of noise in the side-channel traces, under the assumption of weak separability, the low-rank property of the matrix is used to distinguish the signal matrix from the noise matrix; that is, the low-rank Hankel matrix approximation is used to separate the signal and noise. Since SVD is the optimal approximation of matrix in the sense of Frobenius norm, the denoising method using SVD generally decomposes the vector space of noisy signal matrix into signal subspace and noise subspace by truncated singular value decomposition (TSVD). In the preprocessing of side-channel analysis, the trace is embedded into a Hankel matrix one by one, and the original Hankel matrix is decomposed into a low-rank signal matrix and a sparse noise matrix by truncated singular value decomposition, which exploits the low-rank property of the signal matrix. However, when the noise level is high, this signal subspace actually still contains residual noise [13]. Hence, this paper refines three Schatten norm-based hard thresholding truncation methods for fast hard threshold selection, introduces two soft threshold shrinkage damping methods for noise attenuation, and proposes a hybrid threshold denoising framework using singular value decomposition for side-channel analysis preprocessing. The hybrid threshold denoising framework using singular value decomposition is proposed to extract the signal components in the traces and attenuate the residual noise in the signal matrix. The primary contributions of this research can be summarized as follows:

- Three hard threshold calculation methods of truncated singular value decomposition are refined and defined based on the Schatten norm. These hard threshold selection methods are the normalized singular value threshold method, norm ratio singular value threshold method, and contribution rate singular value threshold method;
- Two kinds of singular value soft threshold operations are introduced to shrink and damping singular vectors, respectively;
- A hybrid threshold denoising framework using singular value decomposition based on low-rank matrix approximation (LRMA) theory was proposed by combining singular value hard threshold truncation and singular value soft threshold operation. The hybrid threshold denoising framework included normalized threshold shrinkage, norm ratio threshold shrinkage, contribution threshold shrinkage, normalized threshold damping, norm ratio threshold damping, and contribution threshold damping;
- Two kinds of non-profiled analysis experiments are carried out, which are Correlation Power Analysis (CPA) and Mutual Information Analysis (MIA). In Figure 1, the flow chart of the side-channel analysis based on the hybrid threshold denoising framework using singular value decomposition is given. In this paper, indicators such as signal-to-noise ratio (SNR) and success rate (SR) are used to evaluate the experimental results. The experimental results of CPA and MIA express the effectiveness of the proposed hybrid threshold denoising framework in side-channel analysis preprocessing.

The rest of this article is organized as follows: In Section 2, under the assumption of separability, we provide background information about Hankel matrices and their approximation to low-rank matrices. In Section 3, we discuss the hard thresholding and soft thresholding methods of singular value decomposition in detail for the low-rank Hankel matrix approximation problem. Furthermore, a hybrid threshold denoising framework using singular value decomposition for side-channel analysis preprocessing is proposed based on the Schatten norm. In Section 4, we first describe the experimental configuration and evaluation metrics, and then conduct the non-profiled side-channel analysis experiments on different public datasets, and analyze and summarize the results. The final experimental results verify the effectiveness of the proposed side-channel preprocessing framework. Finally, Section 5 concludes this paper with a summary and outlook.

## 2. Preliminaries

### 2.1. Separability, LRMA, and LRHA

Hassani et al. [14] studied the separability of signal components and noise components in detail and applied singular spectrum analysis (SSA) to solve this problem. Harmouche et al. [15] proposed new theoretical and practical results about separability. Therefore, based on the assumption of separability between signal and noise, we introduce low-rank matrix approximation and use a special structure matrix, namely the Hankel matrix, to effectively disentangle signal and noise components.

The problem of estimating low-dimension subspaces is known as low-rank matrix approximation (LRMA), where the entire high-dimensional data matrix is known and aims to extract its low-rank properties. Low-rank matrix approximation is to find a low-rank matrix A such that the difference E=D−A between the data sample matrix D and low-rank matrix A is minimized, which is defined as follows Equation (1):

(1) $$\min_{A}{∥E∥}_{F}^{2}={∥D-A∥}_{F}^{2}\;s.t.r⩽\mathrm{rank}\left(A\right)$$

where r is is the retained rank and r≪min(m,n). The low-rank matrix can be approximated by decomposing the data matrix into a sum of a low-rank matrix and a sparse matrix, followed by solving an optimization problem for the norm. Therefore, we will introduce the Schatten norm to solve this problem in Section 2.2. When the sparse matrix E follows an independent and identically distributed Gaussian distribution, the problem of approximating a low-rank matrix is transformed into the classical Principal Component Analysis (PCA) problem [16]. LRMA is a widely-used theory for resolving the issue of noise reduction in both signal processing and data enhancement [17]. The present study endeavors to capitalize on this method by initially converting the one-dimensional leakage signal trace into a two-dimensional Hankel time-delay matrix (i.e., Hankelization), and subsequently leveraging the low-rank characteristic of the signal matrix and the sparse nature of the noise matrix to restore the low-rank matrix.

The rank of the Hankel matrix is frequently utilized in control systems theory to characterize the order or intricacy of the corresponding linear dynamical system [18]. The Hankel matrix is a distinctive mathematical matrix whose elements are symmetrically arranged along the anti-diagonal, and it has broad applications in signal processing, numerical calculation, and system control, among other fields. For a given unprocessed side-channel trace $T_{unprocessed}=\left(t_{1},t_{2},\cdots ,t_{N}\right)$, the time-delay Hankel matrix is constructed by selecting an appropriate window length w. The formal structure of the time-delay Hankel matrix H(t) is represented by the following Equation (2):

(2) $$H\left(t\right)=\left[\begin{array}{cccc} t_{1} & t_{2} & \cdots & t_{d}\\ t_{2} & h_{3} & \cdots & t_{d+1}\\ \vdots & \vdots & \ddots & \vdots \\ t_{w} & t_{w+1} & \cdots & t_{N} \end{array}\right]$$

where d=N−w+1 and $2<w\leq \frac{N}{2}$, and the optimal window length can be calculated by the method [19]. Constructing the delay Hankel matrix of one-dimensional side-channel trace is also Hankelization. According to Theorem [20], for a given data matrix, there exists a unique low-rank matrix with a Hankel structure that approximates the original matrix. Therefore, a low-rank Hankel matrix can be used to approximate the original Hankel matrix. The low-rank Hankel matrix approximation (LRHA) problem can be formulated as follows: given a data matrix $D\in R^{m\times n}$, a positive integer k, and a Hankel matrix class $H_{n}$, find a low-rank Hankel matrix $\bar{H}\in H_{n}$ with rank equal to k such that

(3) $$∥D-\bar{H}{∥}_{F}^{2}=\min_{D\in R^{m\times n}}{∥D-H∥}_{F}^{2}\;s.t.H\in H_{n},rank\left(H\right)=k$$

where D is a Hankel matrix, k is the rank of the low-rank Hankel matrix with 1≤r≤rank(D).

Following the hybrid threshold method, the matrix reconstruction of the selected singular value components is performed. It is worth noting that the resulting reconstructed matrix does not conform to the Hankel matrix structure, thus necessitating the application of diagonal averaging. The formula for diagonal averaging is expressed as follows

(4) $${\tilde{t}}_{n}=\left\{\begin{array}{cc} \frac{1}{n}\sum_{i=1}^{n}x_{i,n-m+1} & \mathrm{for}\;1\leq n<W,\\ \frac{1}{W}\sum_{i=1}^{W}x_{i,n-i+1} & \mathrm{for}\;W\leq n\leq D,\\ \frac{1}{N-n+1}\sum_{i=n-D+1}^{N-D}x_{i,n-i+1} & \mathrm{for}\;D<n\leq N, \end{array}\right.$$

where W=min(w,d), D=max(w,d), then the processed side-channel trace $T_{processed}=\left({\tilde{t}}_{1},{\tilde{t}}_{2},\cdots ,{\tilde{t}}_{N}\right)$ is the reconstructed time series.

### 2.2. Schatten Norm

Fazel [21] systematically studied the optimization problem of low-rank matrix for the first time in their doctoral dissertation. Different from the classical continuous optimization model, low-rank matrix optimization problems all contain non-convex and non-continuous rank functions. Low-rank matrix optimization is an NP-hard problem due to the combinatorial property of rank functions [22]. Therefore, the theoretical analysis and algorithm design of such problems will encounter great difficulties. In order to make the low-rank matrix optimization solvable, the rank function of the matrix must be minimized to be relaxed, and this relaxation is related to the Schatten norm, which is defined as follows Equation (5):

(5) $${∥X∥}_{p}={\left(\sum_{i=1}^{min\{m,n\}}\sigma_{i}^{p}\right)}^{1/p}$$

where $\sigma_{i}$ is the singular value of matrix X, and the Schatten norm is equivalent to the p—norm applied to the singular value vector of matrix X.

### 2.3. Singular Value Decomposition

Singular value decomposition (SVD) [23] is a matrix factorization method that can decompose any matrix X into the product of three matrices. It is the optimal approximation of the matrix in the sense of Frobenius norm, which is defined as Equation (6):

(6) $$X=U\Sigma V^{T}=\sum_{i=1}^{r}\sigma_{i}u_{i}v_{i}^{T}$$

where U is an orthogonal matrix of order m, V is an orthogonal matrix of order n, $\Sigma =\mathrm{diag}(\sigma_{1},\sigma_{2},\cdots ,\sigma_{r})$ is a rectangular diagonal matrix of order m×n consisting of non-negative singular values in descending order, and σ is the singular value of matrix X.

The low-rank approximation problem of the Hankel matrix is a complex and difficult problem to solve, but the norm in solving this problem is not required to be F norm. When the matrix norm is F norm, the truncated singular value decomposition (SVD) method can be used to solve it. In addition, singular value shrinkage can be carried out by the OptShrink [24] algorithm, and the weight of the singular value vector can be continuously optimized by the soft threshold, so as to realize low-rank matrix recovery. However, this method requires prior knowledge of the rank of the signal matrix, which is difficult to estimate in practical applications of side-channel analysis.

Therefore, this paper refines three hard threshold calculation methods of truncated singular value decomposition based on the Schatten norm and introduces two singular value soft threshold operations on shrinkage damping. In addition, a hybrid threshold denoising framework using singular value decomposition is proposed by combining hard threshold truncation with soft threshold shrinkage damping, which are normalized threshold shrinkage SVD(NTS-SVD), norm ratio threshold shrinkage SVD(NRTS-SVD), contribution threshold shrinkage SVD(CTS-SVD), normalized threshold damping SVD(NTD-SVD), norm ratio threshold damping SVD(NRTD-SVD), and contribution threshold damping SVD(CTD-SVD), respectively, for the preprocessing of side-channel traces. In this paper, the low-rank matrix approximation theory is used to optimize the rank selection method of singular value decomposition, and the signal-to-noise separation is more effective to improve the side-channel attack performance.

## 3. Methodology

### 3.1. Hard Threshold

Truncated singular value decomposition (TSVD) [25] determines the effective rank by a predetermined hard threshold and truncates the singular value sequence by using the effective rank. The larger singular value is selected for signal reconstruction, and the smaller singular value is set to zero and discarded. This method requires a hard threshold parameter to be defined in advance empirically, and an appropriate parameter selection can produce desirable results. According to the Eckart–Young–Mirsky theorem [26], the optimal solution of Equation (3) can be achieved by truncating the first k relatively large singular values and truncating the smaller ones by the method of truncated singular values, thus completing the low-rank approximation of the matrix. Therefore, based on the Schatten norm, three hard threshold truncation methods of singular value are refined in detail in this paper, which are normalized threshold truncation, norm ratio threshold truncation, and contribution threshold truncation.

#### 3.1.1. Normalized Threshold

The Schatten norm when p=∞ is the same as the spectral norm; that is, ${∥X∥}_{\infty }=\sigma_{\max}\left(X\right)$ is the largest singular value, and the normalized singular value is calculated as follows Equation (7):

(7) $$\eta \left(k\right)=\frac{\sigma_{k}}{{∥X∥}_{\infty }}=\frac{\sigma_{k}}{\sigma_{\max}\left(X\right)}$$

The estimated rank k of the truncated singular value is obtained by selecting the smallest integer satisfying $\eta \left(k\right)\geq \epsilon$, where ϵ is a small positive number, which is chosen based on the precision of the data, the empirical value is ϵ=0.1 or ϵ=0.05.

#### 3.1.2. Norm Ratio Threshold

The Schatten norm when p=2 is equivalent to the Frobenius norm, and the norm ratio calculation is defined as follows Equation (8):

(8) $$v\left(k\right)=\frac{∥X_{k}{∥}_{2}}{{∥X∥}_{2}}=\frac{\sqrt{\sum_{i=1}^{k}\sigma_{i}^{2}}}{\sqrt{\sum_{i=1}^{\min\{m,n\}}\sigma_{i}^{2}}}$$

the estimated rank k of the truncated singular values is obtained by selecting the smallest integer satisfying $v\left(k\right)\geq \alpha$, where α is a threshold close to 1, and the empirical value is α=0.997.

#### 3.1.3. Contribution Threshold

The Schatten norm when p=1 is also known as the nuclear norm and is defined as the sum of all singular values of a matrix. The singular value contribution rate is calculated as follows Equation (9):

(9) $$\varphi \left(k\right)=\frac{∥X_{k}{∥}_{*}}{{∥X∥}_{*}}=\frac{\sum_{i=1}^{k}\sigma_{i}}{\sum_{i=1}^{\min\{m,n\}}\sigma_{i}}$$

the estimated rank k of the truncated singular values is obtained by choosing the smallest integer satisfying $\varphi \left(k\right)\geq C$, where C is the cumulative contribution of singular values, and the empirical value can be C=0.9.

### 3.2. Soft Threshold

#### 3.2.1. Threshold Shrinkage

Singular value thresholding [27] is an iterative algorithm to recover a low-rank matrix by using a convex optimization method that minimizes the nuclear norm. The main idea of the algorithm is that the low-rank estimation matrix of the signal has the same singular value vector as the noisy signal matrix, and the soft threshold is introduced to shrink the singular values. By choosing the appropriate threshold τ, part of the singular values can be effectively contracted to zero to realize the low-rank approximation of the matrix. Primitive matrix singular value of X known threshold value is defined as $\hat{X}=D_{\tau }\left(X\right)={\mathrm{UD}}_{\tau }\left(\Sigma \right)V^{T}$, where $D_{\tau }\left(\Sigma \right)=\mathrm{diag}\left({\left(\sigma_{1}-\tau \right)}_{+},\cdots ,{\left(\sigma_{r}-\tau \right)}_{+}\right)$ for the singular values of soft threshold, ${\left(\sigma_{i}-\tau \right)}_{+}$ is the soft thresholding operation, which is calculated in Equation (10) as follows:

(10) $${\left(\sigma_{i}-\tau \right)}_{+}=\left\{\begin{array}{cc} \sigma_{i}-\tau \;, & \mathrm{if}\;\sigma_{i}>\tau_{{}_{+}}\\ 0\;, & \mathrm{else} \end{array}\right.$$

#### 3.2.2. Threshold Damping

Since the low-rank approximation of the Hankel delay matrix by the truncated singular value decomposition is inevitably affected by the noise component, the result obtained by the truncated singular value decomposition can only be a suboptimal solution to the original matrix approximation. In order to improve the truncated singular value, the singular value damping operation is introduced in this paper, and the damping term $D_{\delta }=\mathrm{diag}\left(\delta_{1},\cdots ,\delta_{r}\right)$ is introduced to damp the residual noise in the singular value component, where $\delta_{i}$ is defined as follows Equation (11), and where β is the damping parameter controlling the attenuation degree of $\delta_{i}$.

(11) $$\delta_{i}=\left\{\begin{array}{cc} 1\;, & \mathrm{if}\;i=1\\ 1-{\left(\frac{\sigma_{i}}{\sigma_{i-1}}\right)}^{\beta }\;, & \mathrm{else} \end{array}\right.$$

### 3.3. Hybrid Threshold

**Figure 2:**
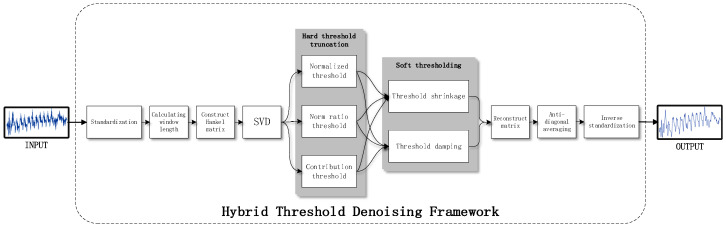
Steps of the hybrid threshold denoising framework.

To preprocess a trace using the hybrid threshold denoising framework, several steps are taken in a specific order. Firstly, the trace is normalized to ensure consistency in scale. Subsequently, the optimal window length is determined using method [19]. The Hankel delay matrix is then constructed based on this optimal window length, and the singular value decomposition of the Hankel delay matrix is performed to obtain the singular value of the trace. The singular values are then subject to hard thresholding, followed by soft thresholding, which shrinks and damping the truncated singular value sequence. The matrix is then reconstructed using the singular values after hybrid thresholding. However, it should be noted that the reconstructed matrix is not Hankel structured, and thus anti-diagonal averaging is performed on it. Finally, the trace is obtained by inverse normalization of the sequence processed by the anti-diagonal average. The detailed steps of the hybrid threshold denoising framework are shown in Figure 2.

#### 3.3.1. Truncated Shrinkage SVD

Singular value decomposition is performed on the Hankelized [28] traces to obtain the singular values $\delta_{i}$ of the matrix. Only k singular values satisfying the hard threshold condition are taken, and the retained singular values are contracted by soft threshold shrinkage operation to make the retained singular values shrink to zero. The left and right singular vector matrices U and V are not changed, only the singular value magnitude is changed. The specific steps of normalized threshold truncated shrinkage SVD, norm ratio threshold truncated shrinkage SVD, and contribution threshold truncated shrinkage SVD are as presented in Algorithms 1–3.
**Algorithm 1** Normalized threshold truncated shrinkage SVD (NTS-SVD)**Input:** Hankelized trace *H*, normalized threshold ϵ **Output:** Normalized threshold truncated shrinkage diagonal matrices $\Sigma_{\tau }$
1:Compute $H=U\Sigma V^{T}$2:Compute the normalized threshold η(k)3:**for** i=1 to *r* **do**4:   $\eta \left(i\right)=\frac{\sigma_{i}}{\sigma_{max}\left(X\right)}$    5:   **if** $\eta \left(i\right)\leq \epsilon$ **then**6:     k=i, $\tau =\sigma_{k}$ and break    7:   **end if**8:**end for**9:Compute the soft threshold shrinkage operator ${\left(\sigma_{i}-\tau \right)}_{+}$10:**for** i=1 to *k* **do**11:   ${\left(\sigma_{i}-\tau \right)}_{+}=\sigma_{i}-\tau$12:**end for**13:Obtain the normalized threshold truncated shrinkage singular diagonal matrix$\Sigma_{\tau }=diag\left({\left(\sigma_{1}-\tau \right)}_{+},\cdots ,{\left(\sigma_{k}-\tau \right)}_{+}\right)$.


#### 3.3.2. Truncated Damping SVD

The singular values $\delta_{i}$ of the Hankelized traces are obtained by singular value decomposition. Only k singular values satisfying the hard threshold condition are taken, and the retained singular values are contracted by soft threshold shrinkage operation to calculate the damping term $D_{\delta }$ so that the retained singular values are attenuated to zero. The left and right singular vector matrices U and V are not changed, only the singular value magnitude is changed. The specific steps of normalized threshold truncation damping SVD, norm ratio threshold truncation damping SVD, and contribution threshold truncation damping SVD are as presented in Algorithms 4–6.
**Algorithm 2** Norm ratio threshold truncated shrinkage SVD (NRTS-SVD)**Input:** Hankelized trace *H*, norm ratio threshold α
**Output:** Norm ratio threshold truncated shrinkage diagonal matrices $\Sigma_{\tau }$
1:Compute $H=U\Sigma V^{T}$2:Compute the norm ratio threshold $v\left(k\right)$3:**for** i=1 to *r* **do**4:   $v\left(k\right)=\frac{\sqrt{\sum_{i=1}^{k}\sigma_{i}^{2}}}{\sqrt{\sum_{i=1}^{min\{m,n\}}\sigma_{i}^{2}}}$    5:   **if** $v\left(k\right)\geq \alpha$ **then**6:     k=i, $\tau =\sigma_{k}$ and break    7:   **end if**8:**end for**9:Compute the soft threshold shrinkage operator ${\left(\sigma_{i}-\tau \right)}_{+}$10:**for** i=1 to *k* **do**11:   ${\left(\sigma_{i}-\tau \right)}_{+}=\sigma_{i}-\tau$12:**end for**13:Obtain the norm ratio threshold truncated shrinkage singular diagonal matrix$\Sigma_{\tau }=diag\left({\left(\sigma_{1}-\tau \right)}_{+},\cdots ,{\left(\sigma_{k}-\tau \right)}_{+}\right)$.


**Algorithm 3** Contribution threshold truncated shrinkage SVD (CTS-SVD)**Input:** Hankelized trace *H*, contribution threshold C
**Output:** Contribution threshold truncated shrinkage diagonal matrices $\Sigma_{\tau }$
1:Compute $H=U\Sigma V^{T}$2:Compute the normalized threshold $\varphi \left(k\right)$3:**for** i=1 to *r* **do**4:   $\varphi \left(k\right)=\frac{\sum_{i=1}^{k}\sigma_{i}}{\sum_{i=1}^{min\{m,n\}}\sigma_{i}}$    5:   **if** $\varphi \left(k\right)\geq C$ **then**6:     k=i, $\tau =\sigma_{k}$ and break    7:   **end if**8:**end for**9:Compute the soft threshold shrinkage operator ${\left(\sigma_{i}-\tau \right)}_{+}$10:**for** i=1 to *k* **do**11:   ${\left(\sigma_{i}-\tau \right)}_{+}=\sigma_{i}-\tau$12:**end for**13:Obtain the normalized threshold shrinkage singular diagonal matrix$\Sigma_{\tau }=diag\left({\left(\sigma_{1}-\tau \right)}_{+},\cdots ,{\left(\sigma_{k}-\tau \right)}_{+}\right)$.


**Algorithm 4** Normalized threshold truncated damping SVD (NTD-SVD)**Input:** Hankelized trace *H*, normalized threshold ϵ, damping parameter β
**Output:** Normalized threshold truncated damping diagonal matrices $\Sigma_{\tau }$
1:Compute $H=U\Sigma V^{T}$2:Compute the normalized threshold η(k)3:**for** i=1 to *r* **do**4:   $\eta \left(i\right)=\frac{\sigma_{i}}{\sigma_{max}\left(X\right)}$5:   **if** $\eta \left(i\right)\leq \epsilon$ **then**6:     k=i and break7:   **end if**8:**end for**9:Compute the soft threshold damping operator $D_{\delta }=diag\left(\delta_{1},\cdots ,\delta_{r}\right)$10:**for** i=1 to *k* **do**11:   **if** i=1 **then**12:     $\delta_{i}=1$13:   **else**14:     $\delta_{i}=1-{\left(\frac{\sigma_{i}}{\sigma_{i-1}}\right)}^{\beta }$15:   **end if**16:**end for**17:Obtain the normalized threshold truncated damping singular diagonal matrix$\Sigma_{\tau }=diag\left(\sigma_{1},\sigma_{2}\delta_{2},\cdots ,\sigma_{k}\delta_{k}\right)$.


**Algorithm 5** Norm ratio threshold truncated damping SVD (NRTD-SVD)**Input:** Hankelized trace *H*, norm ratio threshold α, damping parameter β
**Output:** Norm ratio threshold truncated damping diagonal matrices $\Sigma_{\tau }$
1:Compute $H=U\Sigma V^{T}$2:Compute the norm ratio threshold $v\left(k\right)$3:**for** i=1 to *r* **do**4:   $v\left(k\right)=\frac{\sqrt{\sum_{i=1}^{k}\sigma_{i}^{2}}}{\sqrt{\sum_{i=1}^{min\{m,n\}}\sigma_{i}^{2}}}$5:   **if** $v\left(k\right)\geq \alpha$ **then**6:     k=i and break7:   **end if**8:**end for**9:Compute the soft threshold damping operator $\delta_{i}$10:**for** i=1 to *k* **do**11:   **if** i=1 **then**12:     $\delta_{i}=1$13:   **else**14:     $\delta_{i}=1-{\left(\frac{\sigma_{i}}{\sigma_{i-1}}\right)}^{\beta }$15:   **end if**16:**end for**17:Obtain the norm ratio threshold truncated damping singular diagonal matrix$\Sigma_{\tau }=diag\left(\sigma_{1},\sigma_{2}\delta_{2},\cdots ,\sigma_{k}\delta_{k}\right)$.


**Algorithm 6** Contribution threshold truncated damping SVD (CTD-SVD)**Input:** Hankelized trace *H*, normalized threshold ϵ, damping parameter β
**Output:** Normalized threshold truncated damping diagonal matrices $\Sigma_{\tau }$
1:Compute $H=U\Sigma V^{T}$2:Compute the normalized threshold $\varphi \left(k\right)$3:**for** i=1 to *r* **do**4:   $\varphi \left(k\right)=\frac{\sum_{i=1}^{k}\sigma_{i}}{\sum_{i=1}^{min\{m,n\}}\sigma_{i}}$5:   **if** $\varphi \left(k\right)\geq C$ **then**6:     k=i and break7:   **end if**8:**end for**9:Compute the soft threshold damping operator $\delta_{i}$10:**for** i=1 to *k* **do**11:   **if** i=1 **then**12:     $\delta_{i}=1$13:   **else**14:     $\delta_{i}=1-{\left(\frac{\sigma_{i}}{\sigma_{i-1}}\right)}^{\beta }$15:   **end if**16:**end for**17:Obtain the normalized threshold truncated damping singular diagonal matrix$\Sigma_{\tau }=diag\left(\sigma_{1},\sigma_{2}\delta_{2},\cdots ,\sigma_{k}\delta_{k}\right)$.


## 4. Experiment

Side-channel traces preprocess aims to enhance data features and improve signal quality so as to improve the efficiency of side-channel attacks. In this paper, non-profiled side-channel analysis such as Correlation Power Analysis (CPA) [29] with max discriminant and Mutual Information Analysis (MIA) [30] with cumulative sum discriminant are used to analyze the original traces and the preprocessed traces, respectively. Discriminant is a function that takes as input an array of numbers, applies a specified operation to it, and returns a reduced array. By utilizing this discriminant, we can calculate the key candidate scores through its designated function and converge the results by retaining intermediate values. We adopt signal-to-noise ratio (SNR) [31] and attack success rate (SR) [32] as the primary evaluation metrics, while demonstrating the superiority of our proposed preprocessing framework through maximum correlation value (Table 2) and minimum traces to disclosure [5] (Table 3). Finally, the SCA security metrics and experimental results of the preprocessing are analyzed and summarized.

### 4.1. Preparation

In this work, our experiments were conducted on a computer with Intel(R) Core(TM) i5-9500 CPU 3.00 GHz and 16 GB memory. The preprocessing framework proposed in this paper is implemented using Python 3.8. In order to evaluate the preprocessing effect of the proposed hybrid threshold denoising framework, this paper selects a part of traces from the DPA contest V2 public database [33] and AES_HD dataset [34], which are captured from the FPGA with the noisy. We utilized the Hamming weight (HW) model [35] to represent leakage information for side-channel analysis, while we set experiment parameters ϵ=0.1, α=0.997, C=0.9, and β=3, respectively. Given that the methods proposed in the cited references do not perform well on the datasets used (e.g., EMD methods [7,11]) and some of them are computationally complex and time-consuming, we have included the best-performing wavelet denoising method [6] from the references for comparison.

#### Datasets

**Figure 3:**
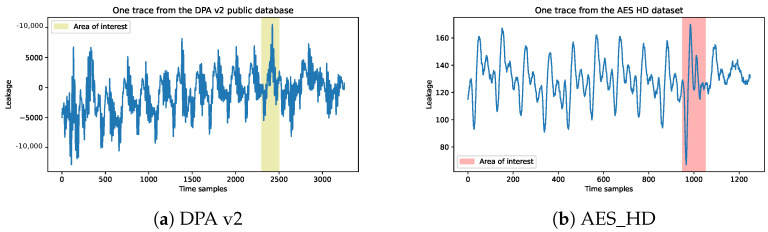
(**a**) One trace from DPA v2 public database with area of interest and (**b**) one trace from AES_HD with area of interest.

- DPA v2: The SASEBO-GII FPGA is employed as the encryption device during signal acquisition in the public database process, operating at a clock frequency of 24 MHz to execute the AES-128 encryption algorithm with stable clock signals. The oscilloscope utilized for signal acquisition boasts a measurement bandwidth of 5 GHz and sampling rate of 5G sampling points per second. Each trace comprises 3253 sampling points, spanning over ten rounds (15.6 clock cycles), with each cycle containing 208.333 sample points, and the collected traces are perfectly aligned. We have selected the final round of AES-128 encryption as our target for attack and collected 100 sample points in each trace for data preprocessing. Below, we display the first trace of the dataset and highlight the area of interest for the attack which corresponds to the last round of the AES-128 encryption, as shown in Figure 3a.
- AES_HD: This is an unprotected AES-128 implemented on FPGA, which was written in VHDL in a round-based architecture that takes 11 clock cycles for each encryption. The AES-128 core is integrated with a UART module to enable external communication, and the design is optimized to expedite measurements and mitigate potential DC offset issues resulting from environmental fluctuations during extended measurement periods. The implementation is realized on a Xilinx Virtex-5 FPGA of the SASEBO GII evaluation board. Side-channel analysis is conducted by capturing electromagnetic radiation emitted from a decoupling capacitor on the power line using a highly sensitive near-field electromagnetic probe. The acquired data are recorded using a Teledyne LeCroy Waverunner 610zi oscilloscope. A total of 500,000 traces are obtained, each corresponding to a randomly generated plaintext and comprising 1250 sample points, as shown in Figure 3b.

### 4.2. Result

**Figure A1:**
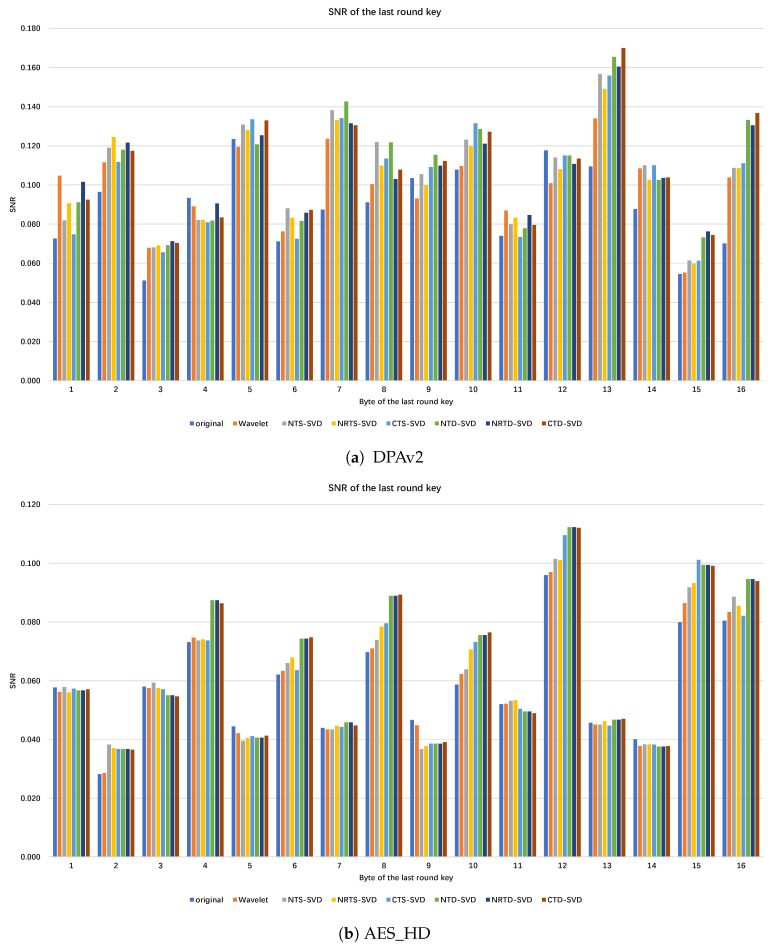
SNR comparison of each byte of AES-128 last round key after using different preprocessing.

In side-channel analysis, SNR is a commonly used evaluation index for preprocessing framework, which reflects the amount of key information that can be obtained from the traces. By calculating the SNR of the correct key for each byte of the AES-128 last round key, as shown in Figure A1, we can conclude that after hybrid threshold denoising framework using SVD for DPA v2, except the 4th byte, the SNR of other bytes increased in different ranges, and the SNR of the 13th byte reached the maximum value. The difference in SNR between different bytes may be caused by the Hamming weight of each byte. The average SNR of proposed preprocessing for all bytes of the last round key increased by 19.70%, 17.01%, 17.21%, 23.16%, 22.39%, and 23.26% respectively, which is better than the wavelet denoising method of 12.29%, as shown in Figure A1a. After applying the hybrid thresholding denoising framework using SVD for AES_HD, the signal-to-noise ratio (SNR) of all bytes except for the 5th, 9th, and 14th byte increased in different ranges, with the SNR of the 12th byte reaching the maximum. The average SNR of proposed preprocessing for all bytes of the last round key increased by 3.68%, 4.87%, 5.83%, 11.01%, 11.01%, and 10.93% respectively, which outperformed the wavelet denoising method by 0.97%, as shown in Figure A1b. The results of SNR show that the proposed preprocessing framework can significantly reduce the noise component in trace and effectively improve the side-channel attack efficiency, and is superior to the wavelet denoising method.

**Figure 4:**
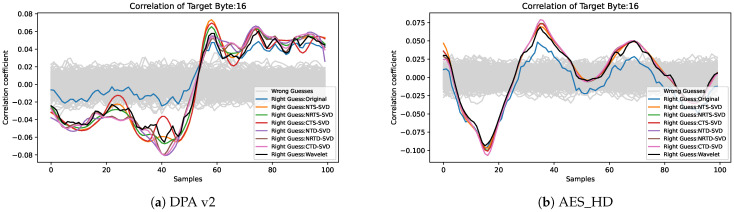
Correlation coefficient comparison of the correct guess key after using different preprocessing: (**a**) the 16th byte of the AES-128 last round key in DPA V2 and (**b**) the 16th byte of the AES-128 last round key in AES_HD.

The correlation coefficient has a positive relationship with SNR, which can also be used as an index to evaluate the efficiency of side-channel attacks. Taking the correlation coefficient curve between the sample point and the correct guess key of the 16th byte of the AES-128 last round key as an example, as shown in Figure 4, the correct guess key and the wrong guess key are clearly distinguished at the peak. After the preprocessing framework proposed in this paper, the distinction between the correctly guessed key and the incorrectly guessed key is more distinct, and the correlation coefficient of the correctly guessed key rises more sharply and has a higher peak. The highest peak of the correctly guessed key curve is shown in Table 2; we can draw a conclusion that the proposed preprocessing framework still outperforms the wavelet denoising [6]. By comparing the data in Table 2, it can be seen that the maximum correlation values after different preprocessing methods have different degrees of improvement. On DPA v2, the soft threshold shrinkage method has a large improvement on the correlation coefficient, and the soft threshold damping method has a small improvement on the correlation coefficient. Compared to the traces without preprocessing, the NTS-SVD improves the correlation coefficient the most by 50.88%, and the NRTD-SVD improves the correlation coefficient the least by 33.71%, both of which are better than wavelet denoising. On AES_HD, the soft threshold damping method has a large improvement on the correlation coefficient, and the soft threshold shrinkage method has a small improvement on the correlation coefficient. Compared to the traces without preprocessing, the NTD-SVD improves the correlation coefficient the most by 63.47%, and the NRTD-SVD improves the correlation coefficient the least by 43.50%, both of which also are better than wavelet denoising.

**Figure 5:**
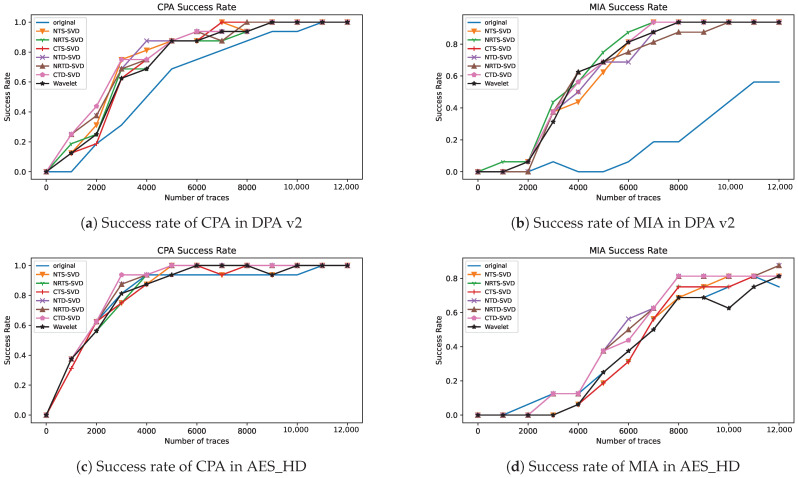
Attack success rate of AES-128 last round key after using different preprocessing: (**a**) success rate of CPA in DPA v2, (**b**) success rate of MIA in DPA v2, (**c**) success rate of CPA in AES_HD, and (**d**) success rate of MIA in AES_HD.

In side-channel analysis, the success rate is another most commonly used evaluation metric, which can fully reflect the efficiency of side-channel attack methods. In order to confirm the effectiveness of the hybrid threshold denoising framework using singular value decomposition proposed in this paper, CPA and MIA are implemented on original traces and the traces preprocessed by the proposed framework. The relationship between the success rate of attack on the last round key of AES-128 and the number of traces is shown in Figure 5. Here, Figure 5a,c is the success rate of CPA, and Figure 5b,d is the success rate of MIA. It can be seen from Figure 5 that using the proposed preprocessing framework, while using the same number of traces, the attack success rate is higher than the original traces, and about 4000 traces are needed to achieve the same attack success rate as the original trace for DPA v2. It can be concluded that the proposed preprocessing framework in this paper can effectively improve the attack efficiency, and the proposed preprocessing operations are overall better than wavelet denoising. However, on the AES dataset, the CPA success rate after the proposed preprocessing method is only slightly better than baseline and wavelet denoising, and the MIA after the proposed preprocessing method is better than baseline and wavelet denoising.

In addition to the above SNR and SR, we can also use the number of traces required to recover a single byte of the key; that is, the number of minimum traces to disclosure (MTD) required for a successful attack to measure the efficiency of the side-channel attack. In this paper, the MTD of the 16th byte of the AES-128 last rounds key is selected to evaluate the preprocessing, and the results are shown in Table 3. The experiment result indicates that MTD is significantly reduced after using the proposed hybrid threshold denoising framework. On the DPA v2, compared to the shrinkage operations, the damping operations perform better at CPA, reducing the MTD by 75% compared to the unpreprocessed traces. In the MID result of MIA, both shrinkage operations and damping operations can reduce MTD by about 50%. Regarding AES_HD, the proposed preprocessing methods exhibit similar or superior performance compared to the baseline. Notably, the performance gain is more prominent in the case of CPA, where the MTD is reduced by almost 50%. However, for MIA, the MTD reduction is only around 10%. In addition, we can also see that the attack efficiency of CPA is much higher than that of MIA, and the difference in attack means will not be discussed here.

## 5. Conclusions

This paper focuses on the preprocessing of traces in the process of side-channel analysis, discusses the rank selection method of hard threshold and soft threshold operations in singular value decomposition, and proposes a hybrid threshold denoising framework using SVD based on low-rank matrix approximation theory for the preprocessing stage of non-profiled side-channel analysis. The experimental results show that the proposed preprocessing framework can better crack the message of the side-channel leakage information hidden in the noise, improve the SNR of the energy traces, greatly reduce the number of traces required for the attack, and is superior to the traditional method in terms of attack efficiency and success rate. In the follow-up study, we will consider extending the proposed preprocessing framework to the domain of profiled side-channel analysis to make it more general. Since the proposed preprocessing framework integrates a variety of threshold selection schemes relying on empirical values, we will continue to optimize the hybrid threshold denoising framework, and a more adaptive rank selection method remains to be carried out in future research work.

## Figures and Tables

**Table 1:** Summary of related works.

References	Filtering Technique	Side-Channel Distinguishers	Security Metrics
[4]	Higher-Order Statistics	DPA, CPA	SNR ^1^, POD ^2^
[5]	Kalman Filter	SPA, DPA, CPA	MTD ^3^
[6]	Wavelet	DPA, CPA	SR ^4^
[7]	EMD-Conv, EMD-IIT	CPA	SNR, SR
[8]	SSA	MCP-DPA, TA	SNR, SR
[9]	Z-score + SVD	CPA	SR
[10]	SSA + DFA + Wavelet	CPA	SR
[11]	EMD-IMFs	CPA	PV ^5^
[12]	Butterworth-LPF + EMD + Wavelet	CPA	CC ^6^

^1^ Signal-to-Noise Ratio; ^2^ Probability of Detection; ^3^ Minimum Traces to Disclosure; ^4^ Success Rate; ^5^ Peak Visibility; ^6^ Correlation Coefficient.

**Table 2:** Comparison of the maximum correlation value of the correct guess key at byte 16 of the AES-128 last round key after using different preprocessing.

Preprocessing Method	DPA v2		AES_HD	
	MCV	Improvement	MCV	Improvement
Original (baseline)	0.049	-	0.048	-
Wavelet [6] (comparison)	0.061	24.55%	0.069	42.20%
NTS-SVD	0.073	50.88%	0.069	43.50%
NRTS-SVD	0.065	34.52%	0.074	52.94%
CTS-SVD	0.070	43.19%	0.073	52.04%
NTD-SVD	0.066	36.17%	0.079	63.47%
NRTD-SVD	0.065	33.71%	0.079	63.46%
CTD-SVD	0.065	34.42%	0.079	63.37%

**Table 3:** After using different preprocessing methods, the CPA and MTD of the 16th byte of the key of the last round of AES-128 are calculated.

Preprocessing Method	DPA v2		AES_HD	
	CPA	MIA	CPA	MIA
Original (baseline)	4350	8550	1272	6130
NTS-SVD	3960	4010	633	5950
NRTS-SVD	3130	4270	627	5950
CTS-SVD	4020	3720	633	5960
NTD-SVD	1220	4010	629	5460
NRTD-SVD	1450	4270	627	5440
CTD-SVD	1240	4000	633	5330

## Data Availability

Publicly available datasets were analyzed in this study. DPA contest V2 public database can be found here: http://www.dpacontest.org (accessed on 1 April 2023) and AES_HD dataset can be found here: https://github.com/AISyLab/AES_HD (accessed on 1 June 2023).
